# Localization and RNA Binding of Mitochondrial Aminoacyl tRNA Synthetases

**DOI:** 10.3390/genes11101185

**Published:** 2020-10-12

**Authors:** Shahar Garin, Ofri Levi, Bar Cohen, Adi Golani-Armon, Yoav S. Arava

**Affiliations:** Faculty of Biology, Technion––Israel Institute of Technology, Haifa 32000, Israel; Shahar.garin@gmail.com (S.G.); Ofrilevi88@gmail.com (O.L.); Bar.cohen711@gmail.com (B.C.); adigol@technion.ac.il (A.G.-A.)

**Keywords:** aminoacyl tRNA synthetase, mitochondria, tRNA, mitochondria import, mitochondria targeting signal, RNA recognition, translation

## Abstract

Mitochondria contain a complete translation machinery that is used to translate its internally transcribed mRNAs. This machinery uses a distinct set of tRNAs that are charged with cognate amino acids inside the organelle. Interestingly, charging is executed by aminoacyl tRNA synthetases (aaRS) that are encoded by the nuclear genome, translated in the cytosol, and need to be imported into the mitochondria. Here, we review import mechanisms of these enzymes with emphasis on those that are localized to both mitochondria and cytosol. Furthermore, we describe RNA recognition features of these enzymes and their interaction with tRNA and non-tRNA molecules. The dual localization of mitochondria-destined aaRSs and their association with various RNA types impose diverse impacts on cellular physiology. Yet, the breadth and significance of these functions are not fully resolved. We highlight here possibilities for future explorations.

## 1. Introduction

Mitochondria are central to cellular physiology, primarily due to their role in maintaining sufficient levels of ATP by the oxidative phosphorylation process. Mitochondria also take important part in additional processes, including calcium and iron homeostasis, production of important cellular metabolites and co-factors and various signaling pathway [1,2,3]. To execute these many functions, approximately a thousand proteins are contained in mitochondria’s different compartments [4,5,6]. Interestingly, while the vast majority of these proteins are encoded in the nuclear genome, translated by cytosolic ribosomes and imported into mitochondria, few proteins (e.g., thirteen in human mitochondria, and eight in yeast) [7] are encoded by the mitochondrial genome. The reasons behind the maintenance of these few genes in the mitochondrial genome, rather than utilization of the host’s nuclear genome are not fully understood. A prominent hypothesis pose that the high hydrophobicity of these proteins may hinder their ability to cross membranes, rendering their nuclear expression less beneficial [8,9]. Another selective pressure is the need for a fast insertion of synthesized proteins into their target oxidative phosphorylation complex [10,11,12]. Also, the sequences encoded inside the mitochondria might turn harmful when expressed at the cytosol, for example due to a resemblance to toxic elements [13] or inclusion of sequences that induce an association with the Signal Recognition Particle (SRP) and mistargeting to the [14].

Regardless of the reason, the expression of these very few mitochondrial genes necessitates the inclusion of a complete translation machinery inside the organelle. This machinery includes mitochondrial ribosomes (with two rRNAs and tens of ribosomal proteins), several translation factors, all types of tRNAs, and the aminoacyl tRNA synthetases (aaRSs) for their charging [15,16,17]. Intriguingly, while all protein components of this mitochondrial machinery are synthesized in the cytosol and imported into the organelle, the RNA components (i.e., rRNA and tRNA) are mostly encoded by the mitochondrial genome. A coordination in synthesis of these compartmentalized components is yet to be established.

## 2. Mitochondrial tRNA Aminoacylation

The aminoacylation of a tRNA by its cognate amino acid is vital for translation. The accuracy and efficiency of this process is essential for proper protein synthesis and hence for cellular viability. tRNA charging with its cognate amino acid is executed by the family of aaRSs; twenty of which are usually present in the cytoplasm, each charges a specific amino acid to all tRNA isoacceptors [18,19]. aaRSs are present also in mitochondria (mt-aaRS), charging their endogenously transcribed tRNAs. This function is also critical for cellular physiology [20,21]. These mt-aaRSs are sometimes encoded by the same genes as the cytosolic ones (dualy-localized mt-aaRS), yet in many cases are encoded by a different gene (exclusively localized). Intriguingly, the extent of the dualy-localized group ranges significantly among species, from almost a complete set (as in *Arabidopsis*) [22], to only two (as in human). Since mitochondrial tRNAs are transcribed from the mitochondrial genome and structurally differ from cytosolic tRNAs [20], exclusively mitochondrial aaRSs may significantly differ from their cytosolic counterparts [23,24]. Beyond the addition of mitochondria-targeting features (described hereafter), mt-aaRSs might have additional features that render them more suitable to mitochondrial tRNAs. For example, anticodon recognition by yeast mitochondrial Threonine tRNA synthetase (ThrRS) is different from its cytosolic counterpart, allowing recognition of the different tRNA^Thr^ [25]. In human mitochondria, SerRS utilizes several unique regions to mediate its association with mitochondrial tRNAs [26]. Thus, mt-aaRSs evolved to utilize different tRNA recognition and charging elements compared to their cytosolic counterparts.

## 3. Protein Import into Mitochondria

**Figure 1:**
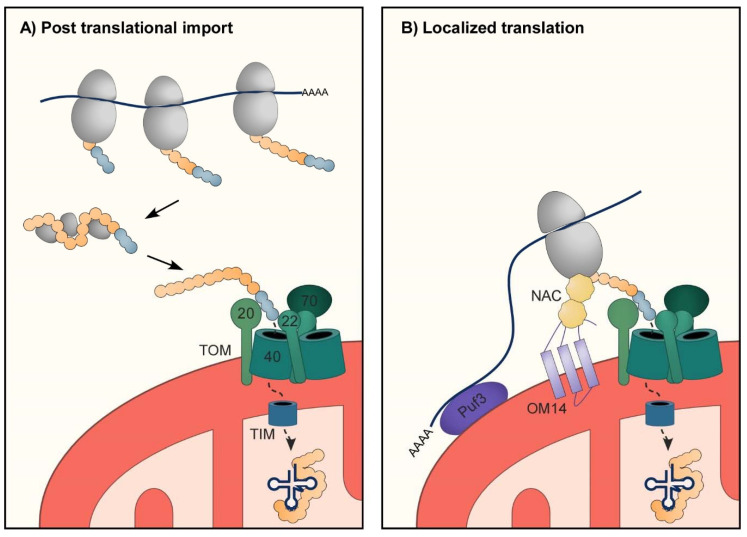
Protein import into mitochondria. (**A**) Mitochondria-destined proteins are translated by cytosolic ribosomes and maintained in an unfolded state by various chaperones. Many of these (including all mitochondrial aminoacyl tRNA synthetases (mt-aaRSs)) bare an N-terminal Mitochondria Targeting Signal (MTS, blue spheres) that enables recognition by protein a receptor on the mitochondria outer membrane (Tom20 for mt-aaRSs). Recognition is followed by insertion through the Tom40 pore and distribution into mitochondria sub-compartments. All mt-aaRSs are transferred through the Translocase of the Inner Membrane (TIM) into the matrix. The MTS of many matrix destined proteins, such as mt-aaRSs, is removed and cleaved by the Mitochondrial Processing Protease (MPP), resulting in a mature, MTS-deficient enzyme. (**B**) Mitochondria proteins can also be imported by a mechanism that involves localized translation near the mitochondria outer membrane [31]. The nascent MTS can interact with Tom20 while the protein is being translated. Furthermore, ribosome-associated chaperones (i.e., Nascent chain Associated Complex (NAC)) can interact with an outer membrane protein (OM14) and support protein import. Finally, the RNA-binding protein Puf3 protein assists in mRNA localization to mitochondria, presumably through interaction with the outer membrane. Notably, while all mt-aaRS mRNAs appear to localize near mitochondria, this localization is only partially affected by Puf3 or Tom20 deletion (Table 1), suggesting a novel mechanism for localization.

Most mitochondrial proteins are encoded by nuclear genes and synthesized by cytosolic ribosomes. After their complete translation, these precursor mitochondrial proteins are maintained in an unfolded form through association with various chaperones, and translocated into the organelle (Figure 1A) [27]. Several pathways of import and sorting into sub-compartments are known today. Each of those pathways involves a different targeting signal within the protein that is recognized by receptors on the mitochondrial outer surface [28,29,30].

The signal for the transport of the vast majority of matrix and inner membrane proteins involves an N-terminal presequence that serves as a mitochondrial targeting sequence (MTS). The MTS is commonly composed of 20–60 amino acids that form a positively charged amphiphilic α-helix structure with a net charge of +3 to +6 [5,35]. This presequence is recognized by protein receptors on the mitochondria outer membrane that belong to the translocase of the outer membrane (TOM) complex.

The TOM complex acts as a main entryway for most mitochondrial precursors (Figure 1A). It is comprised of a few protein components, including a dimer of a β-barrel membrane protein that forms the protein transport channel (Tom40), and three receptor proteins (Tom20, Tom22, and Tom70) [36,37]. Tom20 recognizes the hydrophobic area of the amphipathic presequence, Tom22 receptors, two of which are localized between Tom40 dimer, recognize the positively charged surface, and loosely associated Tom70 acts as a receptor mainly for internal non-cleavable hydrophobic precursors. Following recognition of the MTS by the protein receptor, the mitochondrial protein is transported through Tom40 into the intermembrane space, where it is recognized by the translocase of the inner membrane (TIM) complex. The TIM complex forms the channel into the matrix, through which the precursor protein is transported. Importantly, the presequence translocase-associated motor (PAM) is located at the matrix side and drives the protein import into the matrix. Mitochondria processing peptidases (MPP) are located in the matrix and remove the MTS, thereby generating the mature, functional protein [38]. In recent years, additional import pathways had been described, primarily to target protein into the mitochondria inner and outer membrane or to the intermembrane space [27,30,39]. These are, however, beyond our scope herein since all mt-aaRS appear to be localized in the matrix.

### 3.1. Import of mt-aaRS to Mitochondria

All aaRSs that act in the mitochondria are encoded in the nucleus and need to be imported into the mitochondrial matrix through an MTS mediated pathway [40]. Mutations in MTS of few human mt-aaRSs are implicated in human diseases, in particular neurological disorders, emphasizing the importance of proper import to neuronal function [40,41,42]. For many aaRSs, two genes are present in the genome: one gene has an N-terminal MTS coding sequence which targets it to the mitochondria while the other lacks this sequence and the enzyme is therefore retained in the cytosol. These two genes can be subjected to different evolutionary processes, that adapt each to the compartment it functions at, and to its tRNA target. Interestingly, this concept of one gene–one compartment does not hold for all aaRSs, and in some cases a single gene expresses enzymes for both compartments (the mechanisms enabling this dual localization are described in details below). As a consequence, the same enzyme needs to be functional in both cytosol and mitochondria and recognize diverse types of tRNAs [43]. Whether these aaRSs are more promiscuous in their RNA recognition compared to the compartment-exclusive ones is yet unknown.

**Figure 2:**
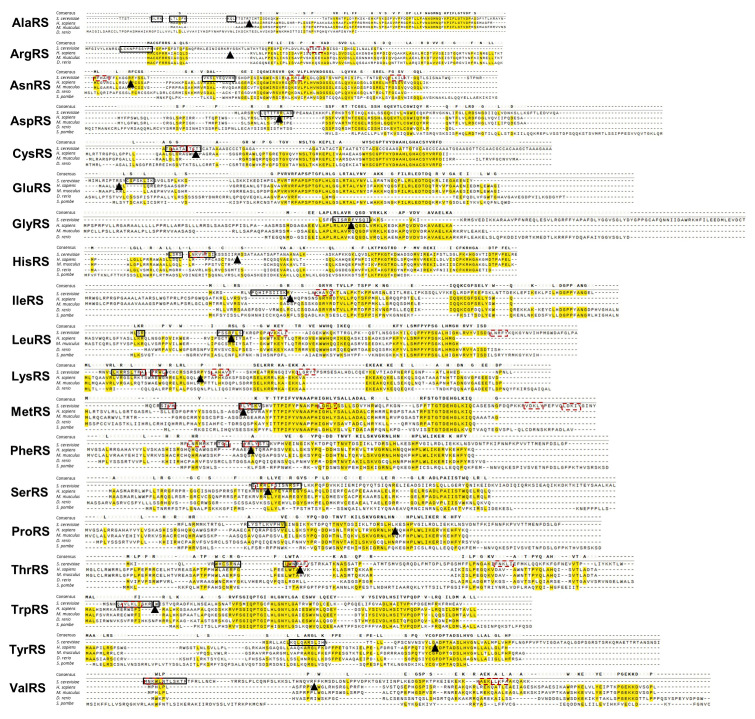
*Saccharomyces cerevisiae* mt-aaRS predicted-MTS and its conservation. The hundred N terminal amino acids of each *S. cerevisiae* mitochondrial aaRS were aligned to their human, *Mus musculus, Danio rerio, Schizosaccharomyces pombe* (when available) orthologs [51]. Consensus sequence is indicated above aligned sequences. Also indicated are the positions of the *S. cerevisiae* amphipathic helix (continues boxes), Tom20 recognition sequence (dashed red boxes) and Mitochondrial Processing Protease (MPP) cleavage site (arrowheads), predicted by MitoFates [45]. Note that MTS sequence conservation is very low and conserved regions are usually functional domains downstream to the MPP. A higher resolution image is provided as a supplementary material (Supplementary Figure S1).

Predictions for MTS of mt-aaRSs from different organisms can be obtained from the MitoMiner web server [44] or MiSynPat [42]. We provide here a compilation of such predictions for *Saccharomyces cerevisiae* mt-aaRSs based on MitoFates web server [45] (Figure 2). Also presented is the conservation of this region compared to few other organisms (Figure 2). Intriguingly, while mitochondria targeting determinants are identified for many of these proteins, their sequence conservation is rather low (Figure 2). Experimental evidence to support the functionality of these regions in import to mitochondria are scarce [46]. e.g., for only six of human mt-aaRSs the targeting peptide was biochemically confirmed (see Table 3 in [47]). Recently, proteomic approaches were developed to better identify mitochondria imported proteins [48] and to define cleaved N termini [35,49,50]. Although these are not exhaustive yet (e.g., only four mt-aaRSs were detected in yeast [35]), they are expected to provide important experimental validation of predicted MTSs.

### 3.2. Mechanisms of Dual-Localization of mt-aaRSs

**Figure 3:**
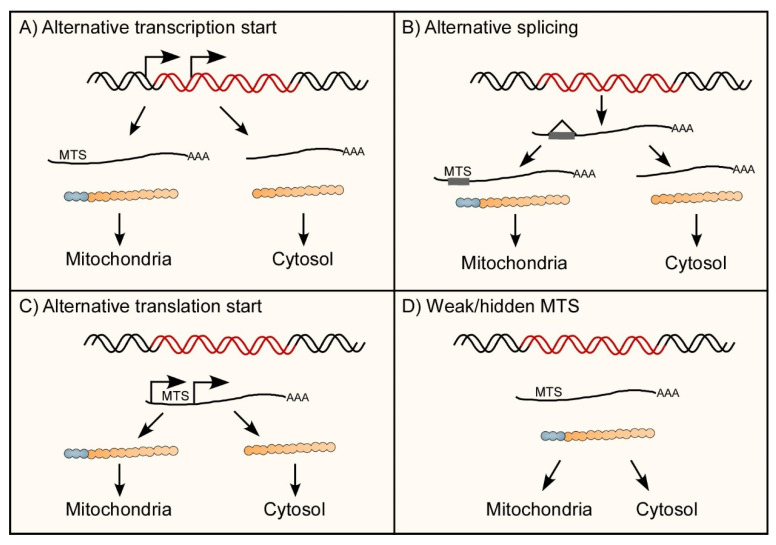
Schematic depiction of mechanisms for aaRS dual targeting. (**A**) A single gene can generate two transcripts by selection of alternative transcription initiation sites. One of the transcripts contains a coding region for an MTS, and leads to synthesis of mitochondria-destined protein, while the other transcript is devoid of this region hence the protein is retained in the cytosol. (**B**) A single pre-mRNA can generate two mature mRNAs by alternative splicing. An exon is retained in one of the transcripts and leads to synthesis of an MTS-bearing protein, which is destined to mitochondria. (**C**) A single mature mRNA can be translated into two proteins, through switching of the translation machinery between two start codons. (**D**) A single translated protein can have a weak or hidden MTS, which leads to partial retention in the cytosol.

Some mt-aaRSs appear to be localized to both cytosol and mitochondria, and charge tRNAs in both compartments. The extent of this duality varies among species, from only two in human, through five in *S. cerevisiae* to almost a complete set in *Trypanosoma brucei* [48] and *Arabidopsis thaliana* [22] (plants are further complexed as some aaRSs also co-localize to chloroplasts). These aaRSs are encoded by a single gene and their dual localization is obtained through various molecular mechanisms that generate two proteins, one with an MTS that is directed to the mitochondria and another without, which is retained in the cytosol (Figure 3). Practically, because in most cases the MTS is cleaved of the enzyme upon entry to mitochondria, the two mature enzymes will be identical.

The following molecular mechanisms were found to underlay dual targeting of mt-aaRSs:

(1) Alternative transcription initiation (Figure 3A): A single gene may be transcribed to several different RNA products by beginning transcription in different sites [52,53]. Different initiation sites create multiple mRNA variants, either including or excluding a coding region for MTS. Few aaRS genes, from various organisms, were found to utilize this route. Few examples were found in *S. cerevisiae*: CysRS gene generates two transcripts, and synthesis of the longer, MTS-containing transcript is coordinated with mitochondrial energy demands through binding of the heme regulatory transcription factor Hap1 [54]. ValRS gene produces two distinct mRNA products. The longer transcript has an additional AUG codon (138 bases upstream to the one in the short mRNA) and mutating this AUG codon induces respiratory deficiency phenotype consistent with a mitochondrial role for this variant. This suggests that the 46 amino-acid peptide encoded by this region serves a mitochondria targeting role [55]. Interestingly, growth complementation assays revealed that incomplete versions of this region are sufficient to complement the growth of a complete deletion of ValRS gene [56]. This rescue of a complete deletion (i.e., of both cytoplasmic and mitochondrial version) by an incomplete MTS region was suggested to be due to inefficient transport of the protein into mitochondria, yielding a dually localized enzyme. This supports the possibility that a weak MTS can underlay targeting to both cellular compartments (see also hereafter). Finally, HisRS gene encodes two distinct transcripts [57]. The longer one (which is transcribed to a much lower levels) includes an addition of 20 amino acids to the protein encoded by the short, abundant isoform. These 20 amino acids have clear MTS features. Mutations in the upstream AUG, or frameshift mutations limited to the predicted MTS, affect mitochondria-related functions (e.g., respiratory growth) and mitochondria targeting of the protein [57,58]. Interestingly, fusion of the first 17 amino acids to LacZ did not lead to mitochondria transport, while the first 53 did. Thus, a full targeting sequence includes the first 20 amino acids of the added peptide, as well as a region from the downstream, cytoplasmic protein. Notably, overexpression of the cytosolic variant improved growth under respiratory conditions [58]. Moreover, a Bi-genomic split GFP (BiG-split GFP) system was recently developed, in which the cytosolic aaRS was fused to a GFP fragment, while the rest of GFP was expressed inside the mitochondria; a clear mitochondrial signal was detected when cytosolic HisRS was tested [59]. This further emphasizes a possible role in mitochondria targeting for a weak MTS that is present in the cytosolic variant.

(2). Alternative splicing (Figure 3B): Splicing is a post transcriptional process that removes parts of the pre-mRNA sequence deemed unnecessary (introns) and assembles the ones needed (exons). In alternative splicing, different sets of exons are selected to create different mRNA products from the same pre-mRNA molecule [60]. These mRNA variants will likely translate different proteins; herein a protein with or without an MTS. For example, two mRNA isoforms are generated by alternative splicing of the first three exons of human LysRS gene. The variant that includes all three is mitochondrial, while the one that includes exons 1 and 3 is cytosolic [61]. It appears that inclusion of exon 2 introduces a stop codon (which terminates any translation that starts in exon 1) and a downstream initiation codon, that leads to translation of a 46 amino acids N terminal addition.

Similar process occurs in the HisRS gene of *Danio rerio*. The gene′s exon 2 is usually spliced out of the mature transcript, creating an mRNA shortened by 309 nts that translates into cytosolic HisRS protein. When exon 2 is retained, a stop codon is introduced (and stops translation from the start codon of exon 1) and a new, exon 2-included AUG is used. This new translation start site results in the introduction of an MTS at the N terminus of the protein and consequently targeting to mitochondria [62].

Finally, in trypanosomatids, almost all aaRSs are dually localized. For IleRS, addition of MTS was shown to occur through a trans-splicing process, which adds a 5′ mRNA extension that includes an MTS. This mechanism is predicted to occur also for AsnRS, ProRS, GluRS and GlnRS [63].

(3). Alternative translation initiation (Figure 3C): The most common mode of translation initiation in eukaryotes is through recognition of the 5′ cap structure, scanning of the 5′UTR and recognition of the first AUG as a translation start site [64,65]. Yet, many exceptions to this mode exist, such as cap independent or non-AUG translation initiation processes [66]. Translation through utilization of alternative start sites was found to be an abundant mechanism for generating two proteins from the same transcript, one with an MTS, destined to mitochondria, and one without that is retained in the cytosol [67]. Many dualy-localized aaRSs exploit such mechanism. For example, a single transcript is transcribed from *S. cerevisiae* AlaRS gene, yet multiple candidate initiation sites are known. Two in-frame ACG codons serve as alternative start codons upstream of the canonical AUG codon. While the short isoform, translated from canonical AUG codon functions as the cytosolic isoform, the sequence added to the long isoform by the redundant upstream ACGs acts as a targeting peptide for mitochondrial import [68].

Similar mechanism underlies the targeting of *S. cerevisiae* GlyRS, where two distinct proteins products are made from a single transcript [69]. The mitochondrial isoform begins translation from a UUG codon, 69 bases upstream of the AUG codon from which the cytosolic enzyme is translated. The added 23 amino acid peptide contains MTS characteristics and acts as a targeting sequence to mitochondrial import. A construct containing the added peptide upstream a different aaRS (ValRS) was able to rescue a mutant lacking the mitochondrial isoform, exemplifying the MTS’s ability to target proteins to the mitochondria [70].

*A. thaliana* provides many examples of dual targeting through alternative translation initiation (of note, in plants aaRSs also need to be targeted to chloroplast by an appropriate signal peptide [22]; this further complexity is discussed in details elsewhere [43]). *A. thaliana* AlaRS mRNA contains two AUG start codons, with the upstream AUG adding 48 aa to the protein product. As a result, two protein isoforms are made from the same transcript, with the shorter (which is commonly more abundant) retained in the cytosol and the longer transported into mitochondria. Fusing the predicted MTS to a reporter gene (plant β-glucuronidase) increased its mitochondrial activity, and replacing the MTS of CoxIV by that of AlaRS allowed for the restoration of respiratory function. These findings suggest that the MTS is functional and sufficient for mitochondrial transport [71]. Similarly, GlyRS, ValRS and ThrRS express a single mRNA with two AUG codons that are used for initiation; the upstream one is used to translate the mitochondrial version of the protein, adding a 39 aa MTS to GlyRS, 56 aa for ValRS and 53 aa for ThrRS. All three peptides have MTS properties, as evident by the mitochondrial localization of GFP fusions of each, and fluorescent microscopy imaging [72,73]. As in most other cases of alternative translation initiation, the downstream AUG appears to be much “stronger” and induce higher expression of the (cytosolic) enzyme. This preferred initiation site is likely due to a better nucleotide environment of this AUG, hence it is more commonly selected by initiating ribosomes [72]. Whether additional features (e.g., IRES elements) are involved in AUG selection is not known.

Recently, human GlyRS gene was found to be transcribed into two mRNA isoforms. Both isoforms contain the information needed for translation of either the cytosolic or the mitochondrial enzymes. However, the long isoform is translated exclusively to the cytosolic enzyme, due to sequence and structure elements that hinder translation initiation from the mitochondrial start codon. The short isoform, on the other hand, can be translated to either the mitochondrial or the cytosolic isoforms of the protein, presumably due to a leaky scanning process that allows selection of both translation start sites [74]. The use of two mRNAs to generate these variants may suggest a yet-to be determined regulatory process.

(4). Weak mitochondrial targeting signal (Figure 3D): Intriguingly, a mitochondrial protein encoded from a single open reading frame (ORF) can be localized to more than one compartment [75] Various mechanisms may underlay the presence of the same protein in the mitochondria and other compartments, including regulated inaccessibility of the signal peptide, the presence of two different signals within a single protein, retrograde movement of the protein after signal cleavage or a weak (ambiguous) signal sequence [76,77]. These mechanisms were shown for plant mt-aaRSs that also present in the chloroplast [78,79,80], yet never formally demonstrated for those that are dually localized to mitochondria and cytosol. Artificial indications for such possibility were obtained by deleting regions of the MTS of *S.cerevisiae* ValRS; some of these incomplete MTSs led to complementation of both cytosolic and mitochondrial ValRS absence. This suggest that weak MTS can complement functions in both compartments [56]. The recently developed BiG-Mito split GFP approach is likely to allow detection of such weak and unexpected MTS sequences, hence expand the list of aaRSs that are dually localized [59].

While mitochondrial tRNA^Glu^ is charged by a mitochondria-exclusive GluRS (encoded by the yeast *MSE1* gene), it was found that small amounts of the cytosolic GluRS (encoded by *GUS1*) can enter the mitochondria by weak targeting sequences pinpointed to the first 30 amino acids of the protein [59] or amino acids 190–199 [81]. The imported cytosolic GluRS can mischarge tRNA^Gln^ with Glu, and this serves as a template for a transamidation reaction that yields a Gln-tRNA^Gln^ molecule (of which no dedicated mitochondrial synthetase is known [82,83]. Notably, the availability of cytosolic GluRS for import is coordinated via its binding to Arc1 protein. When levels of Arc1 decrease (for example, when the yeast grow in a non-fermentable medium), more GluRS is imported to the mitochondria [81]. Thus, Arc1 may mask mitochondria targeting sequences until import is necessary. This suggests a regulatory role for hidden targeting sequences through interactions with protein partners. Interestingly, mitochondrial import of GluRS is coordinated with import of MetRS to the nucleus, altogether regulating expression of genes important for respiratory functions [84].

### 3.3. RNA Localization to Support Import of mt-aaRSs

While it is well established that protein supply to mitochondria is achieved by a post-translational mechanism, primarily by the MTS mediated process described above [29,85], recent studies suggest that RNA localization and localized translation of proteins near mitochondria also support import [31,86] In this process, mRNAs encoding mitochondrial proteins are targeted to mitochondria vicinity and are translated locally to replenish mitochondria with new proteins [32,33,34,87]. Localized translation allows better spatial and temporal regulation of protein synthesis and activity [88,89], and may enable co-translational import of proteins to mitochondria [90,91,92]. Co-translational import prevents the exposure of protein domains to the cytosolic milieu, which may lead to unwanted effects on protein structure and/or cellular physiology [93,94].

RNA localization to mitochondria can be achieved by both translation dependent and independent manner [31] (Figure 1B). Sequence elements that induce RNA targeting to mitochondria are found in the coding region (mostly the MTS) [34] or the 3′ UTR [32,95]. While the nascent MTS interacts with Tom20 protein receptor, 3′ UTR elements interact with RNA binding proteins on the mitochondria outer membrane [32,34,96]. Importantly, a given transcript can harbor targeting elements in both these regions [97,98,99]. Moreover, the localization mode may change during cellular growth or in response to a changing environment [100].

High-throughput studies had identified hundreds of mRNAs in proximity to yeast mitochondria [33,101]. Interestingly mRNAs of all mt-aaRSs that are exclusively localized to mitochondria appeared localized (Table 1). Notably, all these mRNAs behave very similarly, in terms of the contribution of Puf3 to- and the impact of cycloheximide on their localization [32,33] (Table 1). This suggests a mechanism that involves 3′UTR elements and does not involves translation of the MTS. Consistent with this, for these mt-aaRSs which data is available, mRNA localization was not affected from Tom20 deletion [34] Contrarily, localization to mitochondria does not seem to occur for the five mt-aaRSs that are present also in the cytosol (Table 1), which is expected considering the demand for the translated protein in both the cytoplasm and the mitochondria.

Detailed studies on tRNA^Lys^ import to mitochondria revealed that its corresponding synthetase (MSK1) is translated near mitochondria [102]. Although MSK1 is a singular example, and, to the best of our knowledge, the only mt-aaRS whose mRNA localization was specifically analyzed, in light of the genome-wide data we speculate that this phenomenon is relevant to all mt-aaRSs.

## 4. RNA Targets of mt-aaRSs

### 4.1. tRNA Targeting by mt-aaRSs

aaRSs that are localized inside mitochondria charge tRNAs that are used by the mitochondrial translation system. These tRNAs might be transcribed within the mitochondria, from its own genome, or synthesized at the nuclear genome and imported into mitochondria. The extent of organelle-exclusive transcription varies between organisms, from organisms in which a complete set of tRNA is transcribed inside their mitochondria (e.g., *S. cerevisiae*), to others that code none in their mitochondria genome and need to import them all (e.g., Trypanosomatids (see more on tRNA import in [103,104,105]). Regardless of the transcription site of the mt-tRNA, significant differences might occur between the cytosolic and mitochondrial homologs. Thus, mt-aaRSs may apply different interaction modes with tRNA compared to their cytosolic counterparts. This may be of challenge in cases that the same aaRS is present both in the cytosol and mitochondria, and therefore needs to interact with different cognate tRNAs. Whether this is achieved by a lessened binding specificity is yet to be determined.

Specificity of tRNA recognition by aaRSs is determined first by the general, three dimensional tRNA fold (i.e., the three stem-loop RNA structure containing the anticodon, the pseudouridine (Ψ and the dihydrouridine (D) loops). These stem-loops further fold into a ternary L-shape structure that is important for positioning and charging of the tRNA by its aaRS. In addition, specific nucleotides (‘identity elements’) that are located primarily at the anticodon region and acceptor loop serve as a second layer of discrimination. These nucleotides may enhance proper association or inhibit wrong association, and hence are critical for selection of the correct tRNA by its synthetase [106]. Intriguingly, tRNAs encoded by mitochondria genome tend to acquire many mutations compared to their cytosolic counterparts or bacterial genomes [107,108], thus losing many key identity elements [109,110,111,112]. For example, human mt-tRNA^Ala^ lost a key identity element (G3:U70 wobble base pair) that is present in all non-mitochondrial tRNA^Ala^ [113] Another example is mitochondrial tRNA^Asp^, which does not have a G in position 73, a highly conserved identity element for cytosolic tRNA^Asp^ [111,114].

Mitochondrial tRNAs deviate from their cytosolic counterparts not only by point changes in identity nucleotides, but also by including large deletions that affect the general tRNA fold [115,116]. For example, mammalian (human and bovine) tRNA^Ser^ lacks the entire D loop, yet in vitro studies revealed that it can be aminoacylated and is functional in translation [117,118]. Recent sequencing efforts of diverse organisms reveal even greater reductions in mitochondrial tRNAs, up to a complete deletion of both the D and Ψ loops [119,120,121,122,123,124,125,126,127]. While charging of these tRNAs is yet to be validated, the data further challenges standard interaction modes between tRNA and its cognate aaRS. To function properly during translation, these tRNAs need to be recognized properly by their mt-aaRSs and accurately and efficiently charged. Mitochondrial aaRSs, therefore, have relaxed sequence and structural constraints in order to accommodate these needs [20,104]. For example, human mitochondrial AspRS, while highly similar to its bacterial homolog, shows higher structural adaptability to bind non-cognate tRNAs [128]. Human mitochondrial PheRS was found to interact with- and charge tRNA^Phe^ from diverse organisms [129]. This catalytic activity involves significant conformational changes in the active site of the enzyme after substrate binding. Similarly, human mitochondrial AlaRS repurposes protein domains to interact with mitochondrial tRNA^Ala^ that lacks the key G3:U70 identity element [108]. This flexibility in binding is also demonstrated upon docking armless tRNA^Ser^ onto the crystal structure of mt SerRS [26], where multiple noncanonical interactions stabilize binding to this “unnatural” tRNA sequence. Promiscuity is demonstrated also during binding of human mitochondrial LeuRS to tRNA^Leu(UUR)^, which folds into an unusual structure devoid of its D-arm [110]. A final example is from *S. cerevisiae*, where the mitochondrial ThrRS shows unusual flexibility in recognition of its two isoacceptor tRNA anticodon loops, as these differ significantly in their length and sequence [25]. Altogether, these studies iterate the ability of mt-aaRSs to bind non-standard tRNA without impact on charging specificity.

### 4.2. Non-tRNA Targeting by mt-aaRSs

The promiscuity of mt-aaRSs in tRNA binding can lead to interactions with other RNAs. Two mt-aaRSs (LeuRS and TyrRS) are well documented for their interactions with non-tRNA targets. Both mt-aaRSs bind and regulate the splicing of group I introns in the mitochondria. *S. cerevisiae* mt-LeuRS (*NAM2*) directly binds group I introns [130] and assists in their splicing. This interaction is mediated by the mt-LeuRS editing domain (CP1) and have a regulatory role [131]. mt-LeuRS binding is essential for two group I intron splicing targets (bI4 and aI4) and facilitates the splicing of three group I introns (bI2, bI3, and aI3) [132]. Both *Mycobacterium tuberculosis* and human mt-LeuRSs can rescue group I intron splicing in *S. cerevisiae* mt-LeuRS mutants [133]. This suggests a conserved non-canonical function for mt-LeuRS in group I intron splicing activation. Deletion analysis of bI4 intron revealed the region that is active in RNA splicing. This region does not seem to fold into a tRNA^Leu^ like structure [134], indicating a promiscuous mode of binding by mt-LeuRS.

In vitro studies revealed that mt-TyrRS from *Neurospora crassa* is necessary and sufficient for group I intron splicing [135,136]. mt-TyrRS from other Pezizomycotina fungi (*Podospora anserine*) can rescue *N. crassa* mitochondrial group I intron splicing in vitro [137], suggesting a conserved role in splicing for this family. Binding of mt-TyrRS occurs through both its N-terminal catalytic domain and the C-terminal anticodon-binding domain (CTD), with the N-terminus being more important for interaction specificity [136,138,139,140]. Interaction with introns (primarily at the P4-P6 region) is mediated by an RNA-binding surface that is unique to the fungi subphylum Pezizomycotina [141]. This explains why non-tRNA interaction by mt-TyrRS is restricted to these fungi. Importantly, this domain is distinct from mt-TyrR tRNA^Tyr^ binding domain [142], implying that an interaction occurs through the acquisition of new protein domains rather than repurposing existing protein domains [143]. Although bioinformatics structure modeling of group I introns conserved regions (P4–P6) predict tRNA^Tyr^ mimicry [144], the co-crystal structure of mt-TyrRS and P4-P6 did not identify tRNA^Tyr^ identity element [142].

In contrast to the few examples of non-tRNA interactions of mt-aaRSs, a significant body of work shows that cytosolic aaRSs bind non-tRNA targets [145,146,147,148,149,150,151]. Cytosolic aaRSs were found to interact with mRNA and impose important regulatory roles, primarily over the step of mRNA translation. Interactions of these aaRSs with RNA are mediated usually through RNA elements that mimic tRNA identity elements [148,152,153,154,155]. Recent RNA interactome capture studies in *S. cerevisiae* and *Caenorhabditis elegans* revealed that the vast majority of cytosolic aaRSs interact with polyadenylated RNA [156,157]. Unfortunately, these studies were based on polyA selection and therefore did not capture mitochondrial RNA with its associated aaRSs. Thus, the scope of non-tRNA binding by mt-aaRSs is yet to be determined.

## 5. Conclusions

The mitochondrion is a complex and fascinating cellular organelle, likely to originate from an endo-symbiotic process. Perplexingly, mitochondria maintain a complete protein synthesis machinery for the synthesis of a minute fraction of its proteome. Further surprising is the fact that this translation machinery is hybrid, where its RNA components (mRNA, rRNA, and tRNA) are encoded by the mitochondrial genome, and protein components (ribosomal proteins, translation factors and aaRSs) encoded in the nucleus. The protein components are translated in the cytosol and need to be imported to the mitochondria matrix to assemble the functional mitochondrial translation machinery. The mechanism coordinating the supply and assembly of the mitochondria-produced and mitochondria-imported components is yet to be resolved.

aaRSs, the focus of this article, are best known for their role in charging tRNA molecules with their cognate amino acids. From a mitochondria-targeting point of view aaRSs can be divided into two groups: Some of them are exclusively mitochondrial, while others act in both mitochondria and the cytosol (the segregation between these group vary among species). Exclusively mitochondrial aaRSs were shown to utilize different tRNA recognition and charging elements compared to their cytosolic counterparts, rendering them better suited to act upon mitochondrial tRNAs, which differ from their cytosolic counterparts. The second, dualy localized group of aaRSs is intriguing, as it requires the production of two subsets of proteins, that are identical in any other aspect, but that one contains an MTS while the other does not. We described several possible mechanisms to explain this, including alternative transcription initiation, alternative splicing, alternative translation initiation, and the presence of a weak/hidden MTS. We also revealed here an intriguing RNA localization pattern, in which mRNAs encoding exclusively mitochondrial aaRSs are localized and probably translated near the mitochondria outer membrane. This suggests a new mechanism for translation and import of this family of enzymes. All these mechanisms should be thoroughly investigated in order to provide a molecular understanding of the protein factors and RNA elements that mediate proper targeting of mitochondrial aaRSs.

Amounting data suggest additional, non-canonical roles for mt-aaRSs, and aaRSs in general, based on their ability to bind non-tRNA targets. While almost all cytosolic aaRSs were shown to associate with polyadenylated RNA, and were implicated in various translation regulation steps, the few mt-ssRSs that were found to bind non-tRNA targets were involved in splicing regulation. In the future, unbiased experimental methods (e.g., methods that does not rely on polyA selection) would enable a more thorough understanding of the extant of mt-ssRS binding to non-tRNA targets, and its functional significance. Nevertheless, these new findings suggest a novel, non-canonical role of mt-aaRSs in mitochondrial physiology and malfunction.

## Figures and Tables

**Table 1:** Localization of mRNAs encoding mt-aaRSs to the mitochondria vicinity. Data collected from genome-wide RNA localization studies imply localization of mt-aaRS mRNAs to mitochondria vicinity in a translation-independent manner.

Protein Location	aaRS	Yeast Gene	Localization to Mitochondria		Puf3 Dependence ^c^	Translation Dependence ^d^	Tom20 Dependence ^e^
			Biochemical Fractions (MLR Value) ^a^	Ribosome Proximity ^b^			
**Mitochondrial**	ArgRS	*YHR091C (MSR1)*	Yes (36)	Yes	Partial	No	*NA*
	AsnRS	*YCR024C (SLM5)*	Yes (59.8)	Yes	Partial	No	Yes
	AspRS	*YPL104W (MSD1)*	Yes (51)	Yes	Partial	No	Yes
	GluRS	*YOL033W (MSE1)*	Yes (59)	Yes	Partial	No	No
	IleRS	*YPL040C (ISM1)*	Yes (66)	Yes	Partial	No	*NA*
	LeuRS	*YLR382C (NAM2)*	Yes (48.5)	Yes	Partial	No	No
	LysRS	*YNL073W (MSK1)*	Yes (36.7)	Yes	Partial	No	No
	MetRS	*YGR171C (MSM1)*	Yes (63.4)	Yes	Partial	No	*NA*
	PheRS	*YPR047W (MSF1)*	Yes (93.4)	Yes	Partial	No	Yes
	ProRS	*YER087W (AIM10)*	Yes (63.2)	Yes	Partial	No	*NA*
	SerRS	*YHR011W (DIA4)*	Yes (99.1)	Yes	Partial	No	*NA*
	ThrRS	*YKL194C (MST1)*	Yes (72.6)	Yes	Partial	No	*NA*
	TrpRS	*YDR268W (MSW1)*	Yes (87.2)	Yes	Partial	No	No
	TyrRS	*YPL097W (MSY1)*	Yes (94.2)	Yes	Partial	No	No
**Mitochondria & Cytosol**	AlaRS	*YOR335C (ALA1)*	No	No	*NR*	NR	No
	CysRS	*YNL247W (CRS1)*	NA	No	NA	NR	*NA*
	GlyRS	*YBR121C (GRS1)*	Low (13.1)	No	No	NR	No
	HisRS	*YPR033C (HTS1)*	No	No	*NR*	NR	No
	ValRS	*YGR094W (VAS1)*	Low (20.2)	No	No	NR	*NA*

a—Mitochondria localization was determined by fractionation of mitochondria through differential centrifugation, followed by microarray analysis [32]). MLR is the standardized percent of transcripts that were found to be in the mitochondria fraction (indicated in parenthesis). Values > 8% were considered as localized. b—Localization was determined by biotinylation of mitochondria-proximal ribosomes followed by RNA-seq analysis [33]. c—Puf3 dependency was defined as the change in MLR in a *puf3∆* yeast strain compared to its parental strain. “Partial” indicates that the deletion did not fully abolished localization (i.e., MLR is >8% in *puf3∆*) [32]. d—Translation dependency is derived from the impact of cycloheximide on localization of mitochondria-proximal ribosomes [33]. e—Tom20 dependency is defined as a decrease in mRNA localization to fractionated mitochondria by more than two folds upon Tom20 deletion [34]. NA—data non-available, NR—Not Relevant (since the mRNA is not localized).

## Supplementary Materials

The following are available online at https://www.mdpi.com/2073-4425/11/10/1185/s1, Figure S1: Large version of Figure 2
