# Supplementary material for: Localization and RNA Binding of Mitochondrial Aminoacyl tRNA Synthetases
Source: Genes (Basel). 2020 Oct 12;11(10):1185. doi: 10.3390/genes11101185 (PMC7600831; doi:10.3390/genes11101185)
Supplement: Supplementary file 1 [file genes-11-01185-s001.pdf]

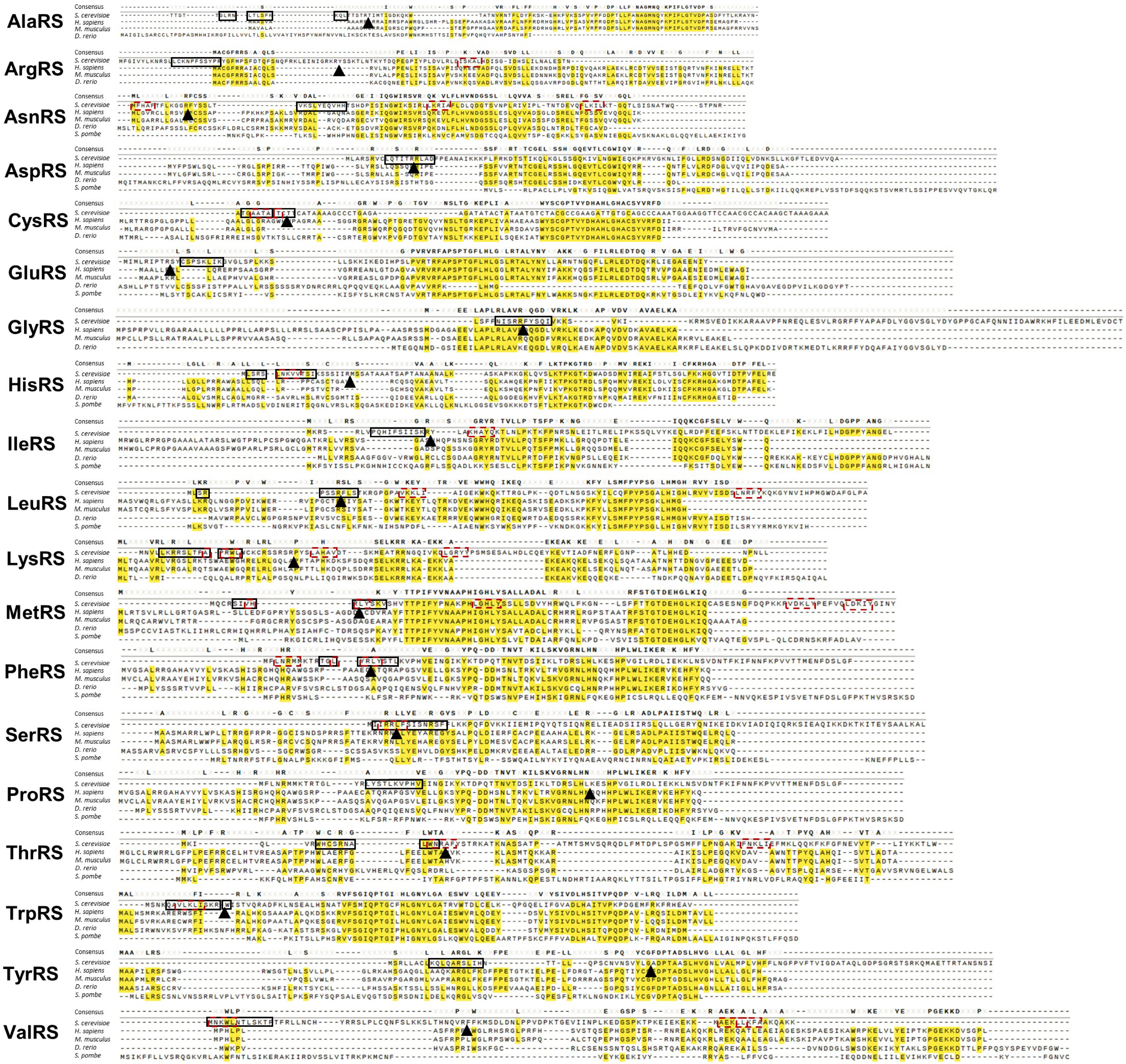
AlaRS
ArgRS
AsnRS
AspRS
CysRS
GluRS
GlyRS
HisRS
IleRS
LeuRS
LysRS
MetRS
PheRS
SerRS
ProRS
ThrRS
TrpRS
TyrRS
ValRS
Consensus
S. cerevisiae
H. sapiens
M. musculus
D. rerio
S. pombe
